# Computing Power Network: Multi-Objective Optimization-Based Routing

**DOI:** 10.3390/s23156702

**Published:** 2023-07-26

**Authors:** Yunpeng Xie, Xiaoyao Huang, Jingchun Li, Tianhe Liu

**Affiliations:** 1 Research Institute China Telecom, Beijing 102209, China; 2 School of Computer Science, Beijing University of Posts and Telecommunications, Beijing 100876, China; ljc@bupt.edu.cn; 3 School of Electronic Engineering, Beijing University of Posts and Telecommunications, Beijing 100876, China

**Keywords:** computing power network, multi-objective optimization, genetic algorithm, NSGA-II, reinforcement learning

## Abstract

This paper presents a novel routing planning method based on multi-objective optimization to tackle the routing problem in computing power networks. The proposed method aims to improve the performance and efficiency of routing by considering multiple objectives. In this study, we first model the computing power network and formulate the routing problem as a multi-objective optimization problem. To address this problem, we introduce a non-dominated sorting genetic algorithm incorporating a ratio parameter adjustment strategy based on reinforcement learning. Extensive simulations are conducted to evaluate the performance of the proposed routing algorithm. The results demonstrate significant client latency and cost reductions, highlighting the algorithm’s effectiveness. By providing a comprehensive solution to the routing problem in computing power networks, this work contributes to the field by offering improved performance and efficiency. The proposed method’s ability to optimize multiple objectives sets it apart from existing approaches, making it a valuable contribution to the research community.

## 1. Introduction

With the global trend towards information and digitization, computing has become an integral part of human life, and the scale of the computing industry has grown exponentially. By 2020, the total global computing power had reached 429 EFLOPS, a measure of the capacity of a device to process data. By 2025, the amount of generated data will reach 80 ZB, and the overall computing power will reach 3300 EFLOPS. Furthermore, the increasing demand for intelligent applications will require more than 1600 EFLOPS of computing power by 2030. However, while intelligent applications have increased the demand for computing power, they have also challenged the supply of computing power, as the need to shorten latency while ensuring throughput has become more pressing [1,2].

To satisfy the computing demands of intelligent services, computing is gradually moving from the cloud to the edge [3,4]. End computing has emerged based on mobile and IoT devices. Hence, computing power is no longer concentrated in data centers but is widely distributed on the edge of the end. As a result, computing power networking is one of the future-oriented development directions for the realization of ubiquitous computing power sharing and scheduling.

The computing power network is the information infrastructure that flexibly allocates and schedules computing, storage, and network resources among clouds, edges, and terminals according to service demands. The computing power network can perceive the information of “computing” and “network” in real-time and provide computing power for services through the orchestration and scheduling of the computing network [5,6,7,8]. Therefore, routing based on service requirements is a crucial issue that needs to be solved urgently [9].

The routing in the computing power network is different from traditional network routing [10,11]. Firstly, traditional routing only considers network attributes. For IP networks, routing only needs to consider bandwidth, such as OSPF or BGP. For optical transport networks, routing needs to consider wavelength and bandwidth. However, whether it is an IP or optical network, routing only needs to consider link states. Computing power network routing needs to consider not only the network attributes but also the computing power attributes, i.e., the computing resources and storage resources. Secondly, traditional routing has clear source and destination nodes, while routing in the computing power network does not contain clear destination nodes [12,13,14,15,16,17]. It is necessary to decide on a suitable destination based on the available resource status.

Currently, computing power network routing is solved as an optimization problem [18], and its optimization objectives primarily focus on a single goal, such as minimizing delay or maximizing network resource utilization [19]. This paper constructs computing power network routing as a multi-objective optimization problem [20,21,22].

In recent years, many studies have combined reinforcement learning with genetic algorithms (GA) to solve practical problems. Chen et al. [23] proposed an optimal RS algorithm based on reinforcement learning using reinforcement learning technology to maintain the diversity of the population in the GA and prevent premature convergence. Wang et al. [24] proposed a multi-strategy selection genetic algorithm based on reinforcement learning to divide the population into three subpopulations for evolutionary purposes, which improves the convergence speed and global convergence problem but only optimizes a single objective. Feng et al. [25] applied the RNSGA-II algorithm that supports reinforcement learning to the multi-objective three-dimensional flight path planning problem in UAVs. By dynamically optimizing migration parameters between populations to maintain population diversity, convergence speed and accuracy improved, but the genetic operation mode only reduced the local search space [26]. To solve the problem of insufficient diversity caused by NSGA-II elite strategies, multiple diversity measures were integrated and reinforcement learning was used to dynamically optimize the split proportion parameters in the iterative process of the population to maintain diversity and improve the convergence performance of the algorithm [27,28,29,30,31]. However, with the Q-learning algorithm, the state and action space is discrete, and the step size of parameter optimization is single, so parameters cannot be changed flexibly, which is not conducive to the fast convergence of the algorithm. In summary, our method addresses the problem of discrete parameter improvement steps in existing reinforcement-learning-enhanced genetic algorithms by combining SAC and NSGA-II. This integration provides a solution for flexible parameter optimization, facilitating faster algorithm convergence.

The significant contributions of our work can be summarized as follows: First, it provides an introduction of the SAC-NSGA-II algorithm as a novel approach to address the routing problem in computing power networks. This algorithm combines genetic algorithms and multi-objective optimization to optimize service performance and resource utilization. It also offers a consideration of the trade-offs between service routing success rate and resource utilization, which previous approaches have overlooked. Our approach aims to balance these factors to achieve optimal routing solutions. Extensive simulations are carried out to evaluate the performance of the proposed algorithm. The results demonstrate the effectiveness of SAC-NSGA-II in reducing service delay and cost while highlighting the trade-offs involved. By providing a concise analysis of related works and highlighting the unique contributions of our study, we aim to address the existing gaps in the literature and contribute to the advancement of routing algorithms in computing power networks.

This paper proposes solving the routing problem based on an RL-enhanced non-dominated sorting genetic algorithm in the computing power network called SAC-NSGA-II algorithms. In this paper, an NSGA-II ratio parameter adjustment strategy based on reinforcement learning was designed. A state space was established according to two measures of population solution spacing and entropy. The soft actor-critic mechanism adjusted the parameters of the population ratio, and the parameters were changed flexibly to adjust the evolutionary direction of the population indirectly to keep the population diversity within a reasonable range. This method can avoid the problem of insufficient diversity caused by the elitist strategy of NSGA-II and its tendency to converge towards the first non-dominated front.

## 2. System Model and Problem Formulation

In this section, we first describe the system model under study in this paper. Then we formulate the problem under study in this paper. Table 1 lists the significant notations used in this paper.

### 2.1. Network Model

The available bandwidth on the link from node *i* to node *j* is denoted as $B_{ij}$ and represents the maximum capacity for data transmission. The traffic from a terminal node is represented by C(s,b,x,y,q,l), where *s* indicates the source node initiating the service, *b* is the requested transmission bandwidth, *x* denotes the required computing resources, *y* represents necessary storage resources, and *q* represents the data size.

For convenience, the computing power network comprises computing power, terminal, access, and routing nodes. In detail, since the cloud and edge computing devices only differ in storage and computing capacity, both are classified as computing power nodes $V_{d},d\in \left[1,D\right]$. Furthermore, terminal devices can be denoted as terminal nodes $V_{s},s\in \left[1,S\right]$, access devices can be denoted as access nodes $V_{a},a\in \left[1,A\right]$, and network devices can be denoted as routing nodes $V_{r},r\in \left[1,R\right]$. Therefore, the computing power network can be formed into a directed graph G(V,E). The set of nodes is represented as $V=V_{d}\cup V_{s}\cup V_{a}\cup$$V_{r}$; the link from node *i* to node *j* is represented as $E_{ij}$, and the set of links composed of all $E_{ij}$ is represented as *E*.

Each computing power node $V_{d}$ also includes certain storage resources and computing resources. $X_{c}$ represents the maximum available computing resources of the $c^{th}$ computing node, and $Y_{c}$ represents the maximum available storage resources of the $c^{th\;}$ computing node. Each link $E_{ij}$ includes a certainly available bandwidth $B_{ij}$, which represents the maximum available bandwidth on the link from node *i* to node *j*

**Figure 1:**
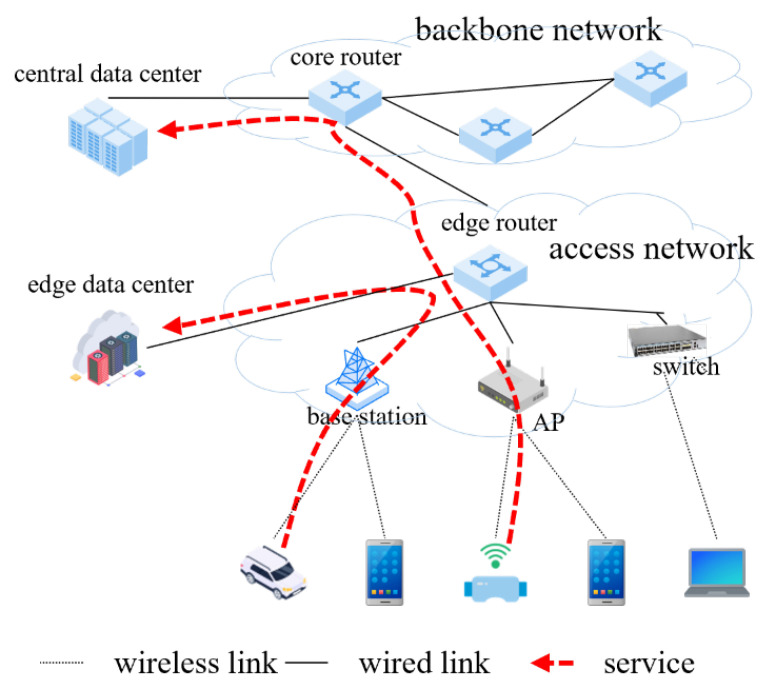
Architecture of the computing power network under study.

The traffic from the terminal node can be C(s,b,x,y,q,l). *s* represents the source node that initiates the service, and *b* represents the transmission bandwidth requested by the service. In addition, *x* represents the size of the computing resources required for the service originating from node *s*; *y* represents the size of storage resources necessary for the service originating from node *s*; *q* represents the size of the data transmitted by the service originating from node *s*; and *l* represents the maximum latency allowed by the service. The architecture of the computing power network under study is shown in Figure 1.

### 2.2. Constraints

In the context of the routing problem in the computing power network, constraints are necessary to ensure the validity of the routed paths for services. This paper classifies the constraints into four distinct aspects, each constraining the relevant variables.

#### 2.2.1. Path-Related Constraints

This paper assumes that the service flow cannot be further divided, so there is at most one transmission path for the $k^{th\;}$ service from the source node *s*. Therefore, Equations (1) and (2) limit the traffic flows outgoing from the source node *s* and incoming to the computing node *d* to 0 or 1. Moreover, the left of the formula indicates whether there is a transmission path for the service, which is $S^{s,k}$.

(1) $$\sum_{j}\lambda_{sj}^{s,k}=S^{s,k},s\in V_{s},k\in \left[1,K\right]$$

(2) $$\sum_{i}\lambda_{id}^{s,k}=S^{s,k},s\in V_{s},d\in V_{d},k\in \left[1,K\right]$$

(3) $$\sum_{d}\sum_{i}\lambda_{id}^{s,k}=S^{s,k},s\in V_{s},d\in V_{d},k\in \left[1,K\right]$$

Because services in the computing power network only originate from the source node, and we assume that the computing power network can only schedule one computing node to serve the service at a time, the $k^{th\;}$ service originating from the source node *s* has only one destination node *d*, as shown in Equation (3).

(4) $$\sum_{i}\lambda_{im}^{s,k}=\sum_{j}\lambda_{mj}^{s,k},s\in V_{s},m\in V_{a}\cup V_{r},k\in \left[1,K\right]$$

Equation (4) shows that the end node of the previous hop is equal to the start node of the next hop of the $k^{th\;}$ service originating from the source node *s* for any intermediate node *m*, where the number of services flowing into or out of *m* is the same to ensure the routed path is continuous.

(5) $$\sum_{i}\lambda_{im}^{s,k}\leq 1,s\in V_{s},m\in V_{a}\cup V_{r},k\in \left[1,K\right]$$

(6) $$\sum_{j}\lambda_{mj}^{s,k}\leq 1,s\in V_{s},m\in V_{a}\cup V_{r},k\in \left[1,K\right]$$

Equations (5) and (6) indicate that for any intermediate node *m*, the times of traversing any intermediate node *m* should not be greater than 1 for the $k^{th\;}$ service originating from the node *s* so that the routed path is not a loop.

#### 2.2.2. Bandwidth-Related Constraints

Equation (7) indicates that the total bandwidth occupied by services on the link $E_{ij}$ should not be greater than the maximum available bandwidth of the link.

(7) $$\sum_{k}\sum_{s}\lambda_{ij}^{s,k}\times \lambda^{s,k}\leq B_{ij},i\in V_{s}\cup V_{r},j\in V_{d}\cup V_{r}$$

#### 2.2.3. Latency-Related Constraints

The latency mainly includes wireless transmission latency, wired transmission latency, calculation latency, and O-E-O conversion latency. The service is initiated from the terminal node to access the base station or the AP through a wireless link, so a wireless transmission delay exists $t_{1}^{s,t}$, as shown in Equation (8).

(8) $$t_{1}^{s,t}=\sum_{j}\lambda_{sj}^{s,k}\times \frac{q^{s,k}*8}{\lambda^{s,k}},s\in V_{s},k\in \left[1,K\right]$$

Afterward, the access network, the cloud network, and the core network are connected by wired links, so there is a wired transmission delay $t_{2}^{s,k}$, as shown in Equation (9).

(9) $$t_{2}^{s,k}=\sum_{i,j}\lambda_{ij}^{s,k}\times \frac{q^{s,k}*8}{\lambda^{s,k}},s\in V_{s},k\in \left[1,K\right]$$

There is a calculation delay $t_{3}^{s,k}$ after the client’s data are uploaded to the computing node, as shown in Equation (10).

(10) $$t_{3}^{s,k}=\frac{q^{s,k}\times \alpha }{x^{s,k}\times 10^{9}},s\in V_{s},k\in \left[1,K\right]$$

Photoelectric conversion is required every time a service passes through a router, so there is still an O-E-O conversion delay $t_{4}^{s,k}$, as shown in Equation (11).

(11) $$t_{4}^{s,k}=\beta \times \left(2\times \sum_{i,j}\lambda_{ij}^{s,k}-1\right),s\in V_{s},k\in \left[1,K\right]$$

Overall, the latency is the sum of the above, which should not be higher than the maximum allowed latency requested by the service, as shown in Equation (12).

(12) $$t_{1}^{s,k}+t_{2}^{s,k}+t_{3}^{s,k}+t_{4}^{s,k}\leq l^{s,k}$$

#### 2.2.4. Computing Resources Constraints

Equation (13) indicates that all computing power resources occupied by clients on the computing node *d* should not exceed the maximum available computing power resources.

(13) $$\sum_{s}\sum_{k}\sum_{i}\lambda_{id}^{s,k}\times x^{s,k}\leq X_{d},d\in V_{d}$$

(14) $$\sum_{s}\sum_{k}\sum_{i}\lambda_{id}^{s,k}\times y^{s,k}\leq Y_{d},d\in V_{d}$$

Equation (14) indicates that all storage resources occupied by clients on the computing node *d* should not exceed the maximum available storage resources.

### 2.3. Optimal Objectives

The optimization model comprises two attributes, namely, computing power and network, and each can be classified into five distinct objectives: latency, cost, routed services, utilization of computing resources, and utilization of storage resources.

#### 2.3.1. Latency

The objective of minimizing the average latency is shown in Equation (15). $t^{s,k}$ is the sum of wireless transmission latency, wired transmission latency, calculation latency, and conversion latency described in Section 2.2.3, and will not be described here.

(15) $$f_{1}=\min\frac{\sum_{s,d}\sum_{k}t^{s,k}}{\sum_{s,d}\sum_{k}S^{s,k}}$$

#### 2.3.2. Cost

Computing power networks prioritize optimizing user satisfaction by reducing client burden costs, which are influenced by varying resource usage costs between computing nodes. To achieve this goal, Equation (16) seeks to minimize the costs associated with using computing and storage resources.

(16) $$f_{2}=\min\frac{\sum_{s,d}\sum_{k}\sum_{i}s^{s,k}k\lambda_{id}^{s,k}\times \left(x^{s,k}\times C_{x}^{d}+y^{s,k}\times C_{y}^{d}\right)}{\sum_{s}\sum_{k}s^{s,k}}$$

#### 2.3.3. Routed Services

Equation (17) represents the maximization of successfully routed services, thereby increasing the number of network-carried services and bolstering network operators’ revenue.

(17) $$f_{3}=\max\sum_{s}\sum_{k}S^{s,k}$$

#### 2.3.4. Computing Resource Utilization

For the computing node *d*, the computing resource utilization is the ratio of all the computing resources occupied by services to the maximum available computing resources. Equation (18) is designed to maximize the utilization of computing resources. In instances where computing nodes are present, idle servers will continue to consume power and energy. This not only fails to generate benefits but also amplifies operating costs. Consequently, optimal utilization of computing resources can effectively augment the revenue of computing nodes, such as data centers.

(18) $$f_{4}=\max\frac{\sum_{s}\sum_{i}\sum_{k}\lambda_{id}^{s,k}\times x^{s,k}}{X_{d}}$$

#### 2.3.5. Storage Resource Utilization

For computing node *d*, the ratio of the storage resources occupied by all services to the maximum available storage resources is the utilization rate of the storage resources. Equation (19) aims to maximize storage resource utilization. Also, this equation can effectively increase the receivables of computing nodes, such as data centers.

(19) $$f_{5}=\max\frac{\sum_{s}\sum_{i}\sum_{k}\lambda_{id}^{s,k}\times y^{s,k}}{Y_{d}}$$

## 3. Proposed SAC-NSGA-II Algorithm

The genetic algorithm (GA) is a commonly used method for solving multi-objective optimization problems. The non-dominated sorting genetic algorithm (NSGA), which utilizes an elite strategy, is particularly well suited for addressing complex multi-objective optimization problems [32]. Therefore, to solve the routing problem, we employ NSGA-II. In addition, we implement a ratio parameter adjustment strategy based on reinforcement learning for NSGA-II. This allows for the flexible adjustment of population ratio parameters through the soft actor-critic mechanism. By indirectly modifying the evolutionary direction of the population, the parameters can be changed to maintain population diversity within a reasonable range.

SAC-NSGA-II comprises four critical operations: encoding and decoding, evaluation, inheritance (crossover, mutation, selection), and a ratio parameter adjustment strategy. To better serve the routing problem of the computing power network, we have redesigned the codec and evaluation operations in this section.

### 3.1. Coding and Decoding

The computing power network’s routing problem aims to determine the transmission paths of services, which must include all service paths. However, using a particular chromosome to represent all paths increases the difficulty of programming. As a result, multiple chromosomes are utilized to represent all paths.

Binary codecs are a more straightforward way to represent paths using chromosomes. Each chromosome consists of multiple genes, representing a node in the network. The value of the gene represents the node through which the path passes. However, this method does not guarantee the path’s legitimacy, as a path may not necessarily be connected, and the value of a gene only indicates whether the path contains the node, not the order of the nodes. The priority codec [33] guarantees the legitimacy of the path, making it suitable for path codecs. Unlike binary codecs, the value of each gene in the chromosome is a non-negative integer, and any two genes have different values, representing the weight of the node included in the path.

However, traditional priority decoding cannot meet the requirements of this paper, as the service in the computing power network cannot give an explicit destination node. Therefore, this paper proposes a priority-based adaptive end-node decoding algorithm (PAEND) based on the traditional decoding method. The pseudocode of the algorithm is shown in Algorithm 1.
**Algorithm 1:** Priority-based Adaptive End-Node Decoding (PAEND).
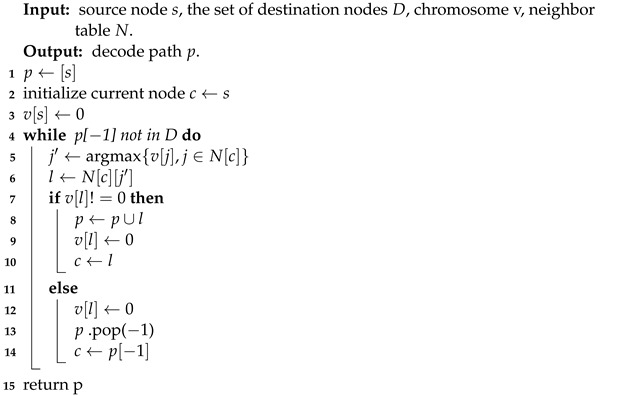


    Among the four input types to the algorithm, the neighbor table data structure is relatively complex, consisting of multiple sets of key–value pairs. The key can be any node in the network, and the value is the set of neighbor nodes corresponding to the key.

PAEND is initialized in steps 1–3, with step 3 assigning the weight of the source node to 0 to prevent forming a loop. In step 4, the algorithm loops to search for the next hop node until the next hop node belongs to the set of destination nodes. In each cycle, the node with the highest weight is selected as the next hop node from the neighbor nodes, as shown in steps 5–6. If node l is visited for the first time, it is included in the path, and the weight of the visited node is set to 0, as shown in steps 7–10. However, if node l is revisited, the algorithm returns the previous node, as the weights of all nodes adjacent to the previous node are also 0, as shown in steps 15–16.

If the destination node cannot be found after traversing all nodes, the algorithm returns to the source node and an empty set is returned, as shown in steps 13–14.

### 3.2. Evaluation

The evaluation function plays a crucial role in determining the search direction and efficiency of the solution and is directly linked to the final solution’s quality. For problems with established mathematical models, the objective function is typically employed as the evaluation function. Therefore, as discussed in Section 2, the evaluation function for the multi-objective optimization routing problem in the computing power network can be constructed using five objective functions. These are minimizing delay, minimizing cost, maximizing the number of routing services, maximizing computing resource utilization, and maximizing storage resource utilization. The pseudocode form of the evaluation function is presented in Algorithm 2.

To initialize the objective function matrix, the number of rows is set to the number of individuals in the population, and the number of columns is set to the number of optimization targets. This matrix is initialized as a zero matrix in step 1. Therefore, the matrix’s $i^{th}$ row records the optimization target value of the $i^{th}$ individual. In step 2, the bandwidth matrix, computing resource matrix, and storage resource matrix are copied since subsequent resource allocation and bandwidth reservation operations will modify the matrix values. In step 16, the original matrix is required to participate in the operation.

The algorithm begins by traversing all individuals in the middle group in step 3. At the start of each traversal, the number of successfully routed services is initialized, and multiple empty sets are created to record the delay and cost of successfully routed services. Next, in step 6, all chromosomes are decoded into service paths through the PAEND algorithm and stored in the set *p*. For each path in $p_{2}$, only the end node in the path that successfully allocates computing resources and storage resources to the service request, and each link on the path that has enough bandwidth reserved for the service, is considered a practical path for the successful routing of the service, as shown in steps 8–11. Among these steps, step 8 aims to transform the path into a structure of node pairs. If the routing is successful, one is added to the number of successfully routed services, and the service transmission delay and cost are recorded in $\underline{\tau }$ and $\underline{c}$, as shown in steps 12–15. When all paths are traversed, in step 16, all objective optimization values are calculated for the $i^{th}$ individual and the objective function matrix is updated accordingly.    
**Algorithm 2:** Evaluation.
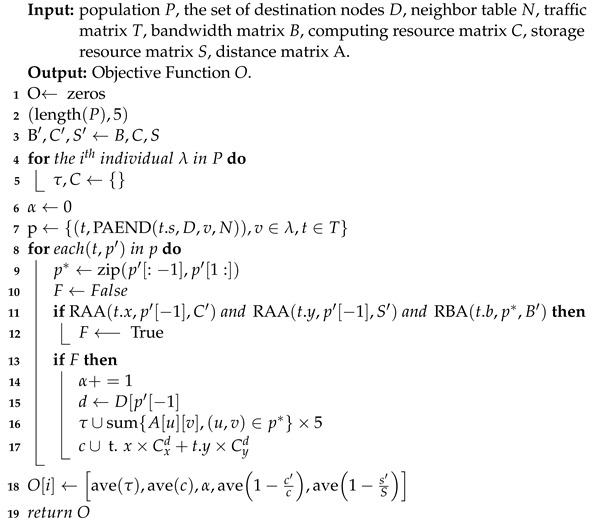


    In step 10 of the evaluation function, a resource allocation algorithm (RAA) and a reserving bandwidth algorithm (RBA) are introduced to judge the validity of the path. The pseudocode of RAA is listed in Algorithm 3, where the resource matrix is 1×N in size, and *N* is the number of computing power nodes. If the destination node has enough resources, it will allocate the requested resource size for the service and return an actual value. The RBA pseudocode is listed in Algorithm 4, where the size of the bandwidth matrix is E×E and *E* is the number of nodes in the network. RBA uses a recursive method to judge whether each hop of the path has sufficient bandwidth from front to back, as shown in step 2. If it is successfully judged to the last hop, the bandwidth will be reserved sequentially from back to front, as shown in steps 4–10. When the bandwidth of any hop link is insufficient, false values are returned sequentially, as shown in steps 3 and 12.    
**Algorithm 3:** Resource Allocation Algorithm (RAA).
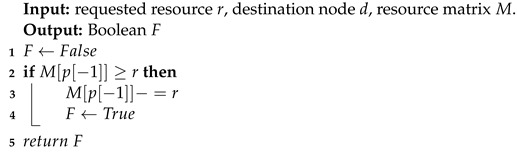


**Algorithm 4:** Reserving Bandwidth Algorithm (RBA).

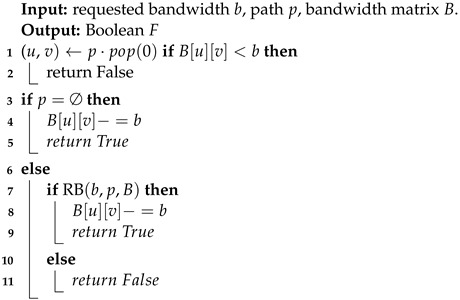



### 3.3. Ratio Parameter Adjustment Strategy

The quality of diversity in the approximate Pareto frontier of a multi-objective problem is directly proportional to the uniformity and discreteness of the non-inferior solution set. Several indicators have been used to measure this quality, including the Sigma, solution spacing, grid, entropy, and individual space metrics. However, relying on a single evaluation index may result in certain deviations. To address this issue, we considered two indices, namely solution spacing and entropy, to evaluate diversity. These indices were combined with soft actor-critic reinforcement learning dynamic control population proportion parameters to optimize the path planning problem of multi-objective computing networks.

#### 3.3.1. Spacing Value

Suppose that the number of Pareto frontier solutions searched by the algorithm is |A|, then the index of solution spacing is defined as

(20) $$S_{p}=\sqrt{\frac{1}{|A|-1}\sum_{i=1}^{\left|A\right|}{\left(\bar{d}-d_{i}\right)}^{2}},$$

where

(21) $$d_{i}=\min\left\{\sum_{h=1}^{H}F_{l}\left(x_{i}\right)-F_{l}\left(x_{j}\right)\right\},i=1,2,\cdots ,|A|,j=1,2,\cdots ,|A|$$

$\bar{d}$ is the mean value of the distance between individuals in the solution set, and *H* represents the number of objective functions. The smaller the solution $S_{p}$, the more uniform the distribution and the better the population diversity.

#### 3.3.2. Entropy Measurement

Suppose the population *X* is divided $X=\left\{X_{1},X_{2},\cdots ,X_{Q}\right\}$, where 1⩽i⩽Q is the partition number, then

(22) $$P_{i}=\frac{\left|X_{i}\right|}{N}$$

where $P_{i}$ represents the probability of an individual falling into the division i; $\left|X_{i}\right|$ represents the number of individuals in the division i;N is the size of the entire population. The calculation formula of population diversity entropy is

(23) $$H=-\sum_{i=1}^{Q}P_{i}\log_{2}P_{i}$$

The higher the entropy, the more discrete and uniform the distribution of individuals in the population, and the better the diversity of the population.

#### 3.3.3. Ratio Parameter Adjustment Strategy

Reinforcement learning is an adaptive optimization control method driven by goals, in which agents interact with the environment to adjust their action strategies. The ultimate objective is to obtain the optimal strategy that maximizes the expected cumulative return.

SAC is an off-policy algorithm that employs stochastic policies. One of its main features is entropy regularization, where the policy is trained to maximize the trade-off between expected returns and entropy. Entropy serves as a measure of the randomness of a strategy. Increasing the entropy also enhances the randomness of the strategy, thereby enabling more exploration, which accelerates subsequent learning. This feature prevents premature policy convergence to the local optimal value.

In NSGA-II, the population is regarded as the agent, and the ultimate goal is proportional parameter learning. The agent senses the change in population diversity and controls the evolutionary direction of the population by controlling the population-proportional parameter. The population-proportional setting is reasonable when the solution spacing decreases compared to the initial population and the entropy measure increases. The state division, action design, and reward mechanism of reinforcement learning are as follows.

State: the state space is composed of a population solution spacing value and an entropy value, $\left[S_{p}^{t},H^{t}\right]$, where $S_{p}^{t},H^{t}$ represent the solution spacing and entropy of the generation population *t*, respectively.

Action: the action of the reinforcement learning agent is the adjustment of the population proportion parameter, $\left[S_{p}^{t},h^{t}\right]$.

Reward: the reward of the agent is determined according to the change in the solution spacing and entropy measure. The goal is to learn the optimal proportion parameter. The specific calculation formula is

(24) $$R=\frac{H_{t}}{H_{0}}-\frac{S_{p}^{t}}{S_{p}^{0}}$$

where $S_{p}^{0}$ and $H_{0}$ represent the solution spacing and entropy of the initial population, respectively.

## 4. Performance Evaluation

This section presents a computing power network and proposes the use of the multi-chromosome multi-objective NSGA-II algorithm (Algorithm 5) to tackle the routing problem. To this end, we first outline the simulation scenarios and parameter settings and subsequently examine the simulation results.
**Algorithm 5:** SAC-NSGA-II Algorithm.**Input**: Network-related and service-related information.**Output**: Routing DecisionInitialize the network and service information and set the algorithm parameters: iteration number *G*, initialize population proportion parameter β, population size *N*, crossover probability $P_{c}$, mutation probability $P_{m}$, reinforcement learning *Q* network, learning rate α and discount rate γThe initial population is generated, and the spacing and entropy measures of the initial population are calculated. Chromosome coding is implemented according to Algorithm 1.Fast non-dominant sorting and crowding degree calculations were performed for the population.Acquire a new population through elite evolutionary strategies.Check whether the maximum number of iterations has been reached. If yes, the iteration is terminated. Otherwise, go to step 6.Calculate the solution spacing and entropy of the population to obtain the state $s_{t}$.Calculate the reward value and update the network parameters of reinforcement learning.Adopt the ε-greedy strategy to select the action $a_{t}$, update population proportion parameters, and go to step3

### 4.1. Simulation Settings

**Figure 2:**
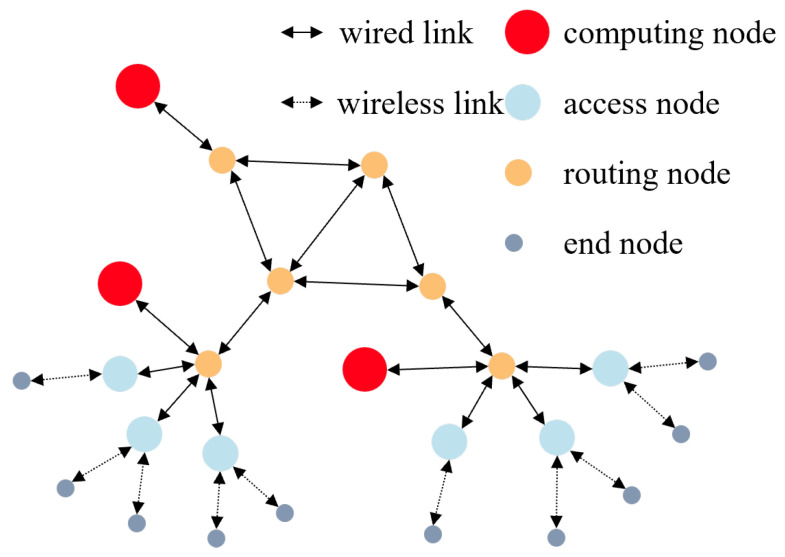
The computing power network used in simulation.

In this paper, we utilize a 25-point network topology, as depicted in Figure 2, for simulation purposes. The topology consists of 6 routing nodes, 6 access nodes, 3 computing nodes, and 10 terminal nodes. The computing power nodes entail computing resources of either 10 or 200 GIPS/CPU and storage resources of 500 GB or 2 TB. The unit cost of computing resources is either 0.1 or 0.6 GIPS, and the unit cost of storage resources is either 0.1 or 0.8 GB. Concerning network links, the wireless link from the terminal node to the access node has a bandwidth of 1 Gb/s, the wired link from the access node to the routing node has a bandwidth of 10 Gb/s, and the wired link connecting routing nodes has a bandwidth of 100 Gb/s. The wired link bandwidth from the routing nodes to the computing power nodes is 40 Gb/s. See Table 2.

Regarding the service, we assume the average requested bandwidth is 300 Mb/s, with an average data volume of 1 GB for single transmissions. The requested computing resources are uniformly distributed between 1 and 20 GIPS, and the requested storage resources are distributed between 1 and 1000 GB. Furthermore, the number of instructions required to process 1 MB of data is 1 GI [34].

The genetic algorithm comprises four essential parameters: the number of populations, the maximum number of generations, the crossover rate, and the mutation rate. In this paper, we set the number of populations to 50 and the maximum number of generations to 300, while the crossover and mutation rates adapt according to the algorithm. Additionally, we take the average value of each set of data repeated 10 times as the final result.

### 4.2. Simulation Results

This section presents the simulation results of the SAC-NSGA-II algorithm, which is multi-chromosome and multi-objective, and varies with traffic load. We analyze the learning curve and fitness curve of SAC-NSGA-II, respectively. Additionally, we compare the spacing value and entropy measures between the SAC-NSGA-II and NSGA-II algorithms. Furthermore, this section compares and analyzes the performance of the algorithm in various aspects, such as success rate, delay, and cost, using benchmarks such as the Dijkstra algorithm (referred to as the D algorithm), soft actor-critic (SAC), Deep Q Network (DQN), and NSGA-II.

#### 4.2.1. Analysis of Algorithm Convergence

**Figure 3:**
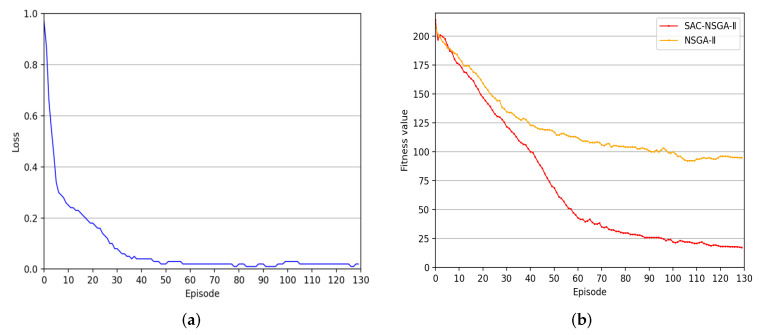
Algorithm convergence of SAC-NSGA-II: (**a**) Learning curve of SAC-NSGA-II. (**b**) Comparison of fitness curves.

Based on Figure 3a, the algorithm exhibits efficient and rapid convergence. It reaches a stable convergence state after 50 steps. Figure 3b presents a comparative analysis of fitness curves, illustrating the significant enhancement in the convergence rate of NSGA-II through the adoption of the proportional parameter adjustment strategy based on reinforcement learning. This strategy effectively addresses the issue of NSGA-II’s tendency towards local convergence, resulting in a faster convergence rate and improved population fitness value.

Furthermore, it is important to analyze the algorithm’s complexity and provide insights into the running time of the proposed algorithm under the specific simulation platform. Our findings demonstrate that our method outperforms traditional approaches regarding convergence speed and overall effectiveness, even though it may require a certain computational cost. The accelerated convergence effect achieved by our approach outweighs the computational overhead, making it a more efficient solution.

#### 4.2.2. Population Diversity Measurements

**Figure 4:**
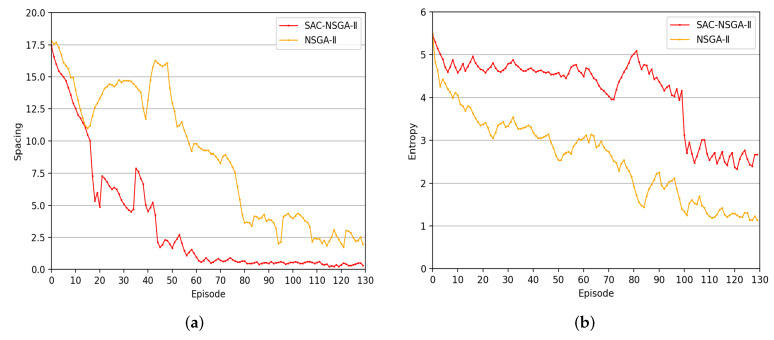
Learning curves of the two diversity measures: (**a**) Comparison of solution spacing values. (**b**) Comparison of entropy measures.

Regarding the learning curves in Figure 4a,b, the rapid decrease in and maintenance of spacing values within a small range after the implementation of SAC-NSGA-II indicate the effectiveness of the proposed approach in enhancing population uniformity. This suggests that SAC, as an additional component, contributes to improving diversity preservation compared to the standard NSGA-II algorithm. The ability to maintain better population entropy measures over a wider range during the iterative process further supports the conclusion that SAC-NSGA-II can effectively enhance population diversity.

The improved diversity preservation achieved by SAC-NSGA-II has important implications. It helps to ensure that the obtained solution set covers a larger portion of the Pareto front, providing more options for decision makers. A diverse set of solutions allows decision makers to explore a broader range of trade-offs between conflicting objectives, thus offering more flexibility in decision-making processes.

Moreover, the ability of SAC-NSGA-II to maintain higher population entropy measures throughout the iterative process suggests that it can effectively handle complex optimization problems with non-linear and non-convex Pareto fronts. By maintaining a diverse set of solutions over a broader range, SAC-NSGA-II enhances the chances of finding globally optimal or near-optimal solutions that may exist in different regions of the Pareto front.

Overall, the results demonstrate the superiority of SAC-NSGA-II in preserving population diversity and expanding the exploration capability of the algorithm. Including SAC as a supplementary strategy in NSGA-II effectively tackles the issue of elitism-related limitations. It enhances the algorithm’s overall performance in obtaining a well-distributed set of solutions on the Pareto front.

#### 4.2.3. Success Rate

**Figure 5:**
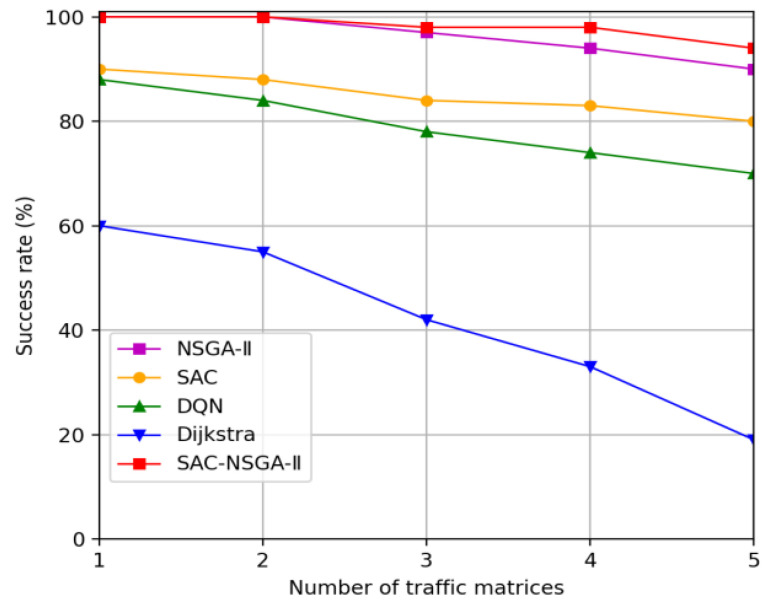
Service routing success rate versus traffic matrix.

The analysis of Figure 5 reveals exciting trends in the routing success rate among different methods as the number of traffic matrices increases. It is observed that the routing success rate decreases for all methods with an increase in demand, indicating the network’s limited capacity to meet the routing requirements of all tasks.

However, SAC-NSGA-II, which incorporates link and node computing resource considerations, achieves the highest routing success rate. This can be attributed to the ability of SAC-NSGA-II to optimize resource allocation and handle the increased demand more efficiently. By considering the availability of link and node resources, SAC-NSGA-II can better distribute and allocate resources to satisfy the routing requirements, resulting in a higher success rate.

In contrast, the NSGA-II, SAC, and DQN algorithms exhibit lower success rates. This could be attributed to the challenges that reinforcement learning algorithms, such as DQN, face in converging to the optimal solution. The complex and dynamic nature of routing problems, coupled with the ample search space, can hinder the ability of reinforcement learning algorithms to find the best routing configurations consistently.

Interestingly, Algorithm D, which only considers link bandwidth availability, during routing, exhibits the lowest success rate. This could be attributed to the excessive resource allocation resulting from the lack of consideration for node computing resources. The inefficient allocation of resources can lead to congestion and potential routing failures.

Overall, the results suggest that incorporating resource availability considerations, as performed in SAC-NSGA-II, can significantly improve the routing success rate, making it a more suitable approach for increasing demand and optimizing resource allocation in network routing scenarios.

#### 4.2.4. Cost

**Figure 6:**
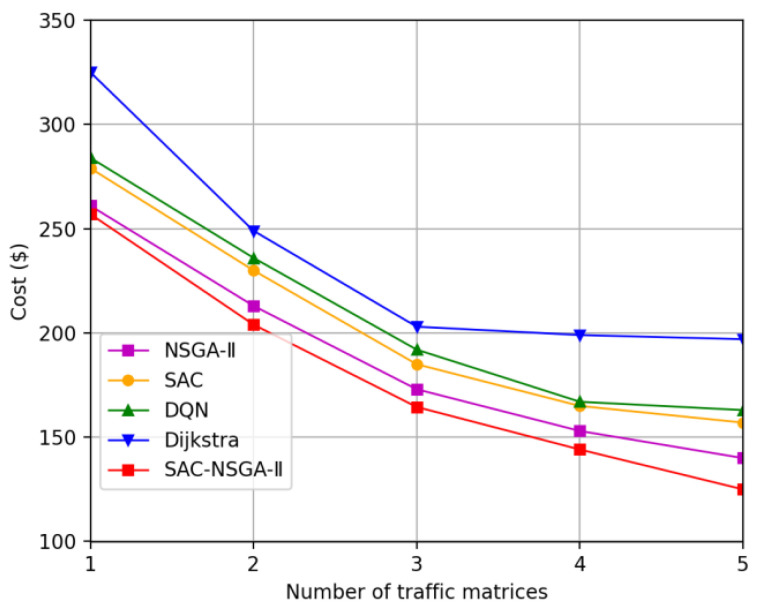
Service average cost versus traffic matrix.

Figure 6 presents the variation in the average single service cost with the increasing number of traffic matrices. Notably, Algorithm D demonstrates the smallest service cost gap compared to SAC, DQN, NSGA-II, and SAC-NSGA-II when three traffic matrices are considered.

This can be attributed to the specific scenario where each terminal node contains three service requests, and the average bandwidth per service request is 300 Mb/s. In this case, the total bandwidth required for allocation is 900 Mb/s. However, the wireless link connecting the terminal node and the access node has a limited transmission bandwidth of 1 Gb/s. As a result, the service request bandwidth nearly reaches the upper limit of the link’s transmission capability.

When the number of input traffic matrices exceeds three, the bandwidth utilized by a single user exceeds the maximum transmission bandwidth, leading to link congestion. In such situations, SAC, DQN, NSGA-II, and SAC-NSGA-II prioritize routing low-cost services, with SAC-NSGA-II exhibiting the lowest cost among them.

It is worth noting that Algorithm D does not actively optimize cost and shows slow changes after the third point. This could be due to the algorithm’s static nature, as it only considers link bandwidth availability during routing without actively optimizing cost.

By contrast, the SAC-NSGA-II algorithm effectively reduces the average single service cost compared to other benchmark algorithms. This can be attributed to the optimization capabilities of SAC-NSGA-II in considering the limited transmission capacities of links and mitigating potential congestion issues as the demand increases. Balancing cost and resource constraints enables SAC-NSGA-II to achieve lower service costs with improved efficiency.

Overall, the results highlight the advantages of SAC-NSGA-II in optimizing service costs while considering various constraints. The algorithm effectively manages the limited transmission capacity of links and dynamically adjusts resource allocation to mitigate congestion issues, leading to more cost-effective solutions compared to other benchmark algorithms.

#### 4.2.5. Latency

**Figure 7:**
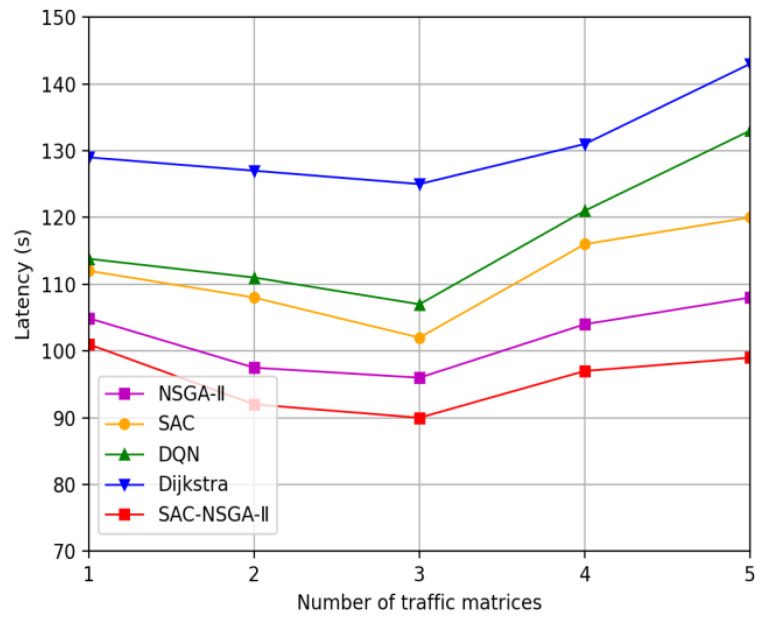
Service average latency versus traffic matrix.

Based on Figure 7, we can observe the trend of service delay as the number of traffic matrices increases. Initially, the service delay is relatively high with a small number of input flow matrices. However, as more traffic matrices are introduced, the service delay decreases.

This decrease in service delay can be attributed to the presence of redundant link bandwidth. With more traffic matrices, there is a better distribution of traffic, leading to improved traffic flow and reduced waiting times for service. As a result, the overall service delay decreases.

However, congestion occurs between the wireless link and the network once the number of traffic matrices exceeds three. This congestion causes an increase in service delays. The limited capacity of the wireless link, combined with the increasing traffic load, leads to service delivery delays.

Interestingly, when three traffic matrices are input, the SAC-NSGA-II algorithm demonstrates a minimum service delay of 90.8 s. This is only 7.9 s slower than the second-best-performin NSGA-II algorithm. This highlights the effectiveness of the SAC-NSGA-II algorithm in optimizing service delay.

Moreover, when the number of traffic matrices increases to five, the maximum service delay reaches 99.4 s. This is 9.6 s slower than the second-best NSGA-II algorithm. This indicates that the SAC-NSGA-II algorithm performs better in managing the increasing traffic load than other algorithms.

In summary, the analysis of Figure 7 reveals that all algorithms exhibit the minimum service delay when inputting three flow matrices. This suggests that three traffic matrices provide an optimal balance between traffic distribution and congestion avoidance. As the number of traffic matrices increases beyond this threshold, congestion occurs, resulting in increased service delay. The SAC-NSGA-II algorithm stands out for its efficient optimization of service delay, making it a promising choice for managing network traffic.

#### 4.2.6. Resource Utilization

**Figure 8:**
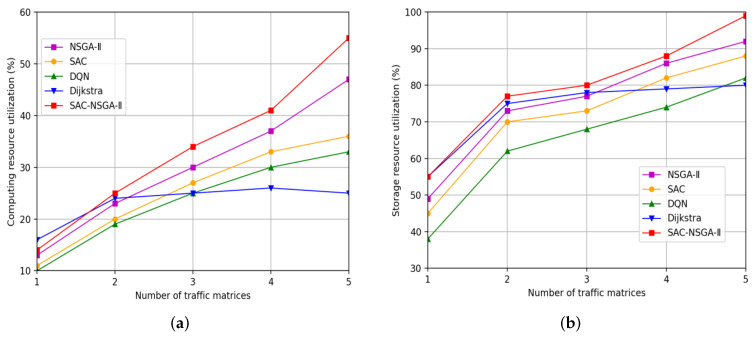
Resource utilization versus traffic matrix: (**a**) Computing resource utilization versus traffic matrix. (**b**) Storage resource utilization versus traffic matrix.

The results presented in Figure 8 offer valuable insights into computing and storage resource utilization in response to the traffic load.

Analyzing Figure 8a, it is observed that Algorithm D exhibits the highest computing resource utilization when only one traffic matrix is considered. This can be attributed to the algorithm’s approach of allocating computing resources based on link bandwidth availability during routing. However, as input traffic matrices increase, SAC-NSGA-II outperforms Algorithm D and maximizes computing resource utilization. This indicates that SAC-NSGA-II is more effective in dynamically allocating computing resources based on evolving traffic conditions. Furthermore, the observation that each additional traffic matrix leads to an approximately 8% DIF > increase in computing resource utilization highlights the growing demand for computational resources in network scenarios with higher traffic loads.

Turning to Figure 8b, the variation in storage resource utilization is depicted. Algorithm D exhibits the highest storage resource utilization when only one traffic matrix is considered. This can be attributed to its static allocation strategy, prioritizing storage availability over other factors. However, the DQN algorithm demonstrates the lowest utilization rate, significantly lower than that of Algorithm D. This indicates that the DQN algorithm struggles to allocate storage resources based on the changing traffic conditions effectively. Conversely, when the number of input traffic matrices exceeds one, SAC-NSGA-II leverages computing resources to achieve maximum storage resource utilization. This suggests that SAC-NSGA-II dynamically optimizes storage resource allocation to manage network congestion better and adapt to changing traffic patterns.

In summary, the computing and storage resource utilization analysis highlights the advantages of SAC-NSGA-II in optimizing resource allocation in response to varying traffic loads. By dynamically adjusting computing and storage resource allocation based on real-time conditions, SAC-NSGA-II demonstrates higher utilization rates and better adaptability than other algorithms. These findings underline the potential of SAC-NSGA-II in improving network performance and resource efficiency in complex network scenarios with traffic variability and congestion challenges.

## 5. Conclusions

In conclusion, this paper has addressed the routing problem in computing power networks by introducing a novel approach based on multi-objective optimization. The study begins by presenting the architecture of computing power networks and constructing a computing power network and service model. Leveraging these models, the routing problem is transformed into a multi-objective optimization problem, and a corresponding model is developed. Recognizing the inherent difficulty of solving multi-objective optimization problems, this paper proposes the SAC-NSGA-II algorithm as a solution. Through extensive simulations, the performance of the proposed algorithm is evaluated, and the results are reported. These results reveal that the genetic algorithm effectively reduces service delay and cost. However, it is important to consider the trade-offs associated with this improvement. Specifically, a slight decrease is observed in both the success rate of service routing and the utilization rate of various resources. This study contributes to understanding the routing problem in computing power networks by providing these findings. The proposed SAC-NSGA-II algorithm offers a promising solution for optimizing service performance regarding delay and cost. However, it is crucial to carefully consider the trade-offs between service routing success rate and resource utilization. Future research directions may involve further optimizing the SAC-NSGA-II algorithm to mitigate the observed trade-offs and enhance overall system performance. Additionally, investigating alternative approaches to address the routing problem in computing power networks could provide valuable insights for further advancements in this field.

## Figures and Tables

**Table 1:** Major Notations Used.

Notations	Definitions
*V*	The set of network vertexes.
$X_{c}$	The maximum available calculating resources in the $c^{th}$ computing node.
$Y_{c}$	The maximum available storage resources in the $c^{th}$ computing node.
$C_{x}^{c}$	The cost of using one GIPS calculating resource in the $c^{th}$ computing node.
$C_{y}^{c}$	The cost of using one GB storage resource in the $c^{th}$ computing node.
*E*	The set of network edges.
$B_{ij}$	The maximum available bandwidth in edge $E_{ij}$.
*K*	The number of traffic matrices.
$T_{k}$	The $k^{th}$ traffic matrix, where k∈[1,K]. Services originate from terminal nodes and there are no destination nodes.
$\lambda^{s,k}$	The requested bandwidth by the node *s* in the $k^{th}$ traffic matrix, where the unit is Gb/s.
$q^{s,k}$	The requested data by the node *s* in the $k^{th}$ traffic matrix, where the unit is GB.
$x^{s,k}$	The requested calculating resources by the node *s* in the $k^{th}$ traffic matrix, where the unit is GIPS.
$y^{s,k}$	The requested storage resources by the node *s* in the $k^{th}$ traffic matrix, where the unit is GB.
$l^{s,k}$	The allowed latency requested by the node *s* in the $k^{th}$ traffic matrix, where the unit is second.
α	The number of IPS when calculating 1GB data.
β	The O-E-O transform latency.
$\lambda_{ij}^{s,k}$	$\lambda_{ij}^{s,k}\in \left\{0,1\right\}$. The service originating from node *s* in the $k^{th}$ traffic matrix and employs the edge $E_{ij}$ as an intermediate segment. If $E_{ij}$ is a path segment, $\lambda_{ij}^{s,k}=1$; otherwise, $\lambda_{ij}^{s,k}=0$
$S^{s,k}$	$S^{s,k}\in \left\{0,1\right\}$. Whether the service originating from node *s* in the $k^{th}$ traffic matrix is successfully routed. If routed successfully, $S^{s,k}=1$; otherwise, $S^{s,k}=0$.

**Table 2:** Simulation parameters.

Parameters	Description	Values
$B_{wd}$	Wired link bandwidth	10 Gb/s, 40 Gb/s, 100 Gb/s
$B_{w}l$	Wireless link bandwidth	1 Gb/s
$S_{d}$	Service request data size	1 GB
$S_{b}$	Service request bandwidth	300 Mb/s
$S_{cpu}$	Services request computing resources	[1 GIPS, 20 GIPS]
$S_{sto}$	Services request storage resources	[1 GB, 1000 GB]
$N_{irpd}$	Number of instructions required per MB of data	1 GI
$C_{cpu}$	Computing resource cost	0.1, 0.6 USD/GIPS
$C_{sto}$	Storage resource cost	0.1, 0.8 USD/GB
$S_{c}^{r}$	Size of edge/central cloud computing resources	10/200 GIPS per CPU
$S_{e}^{r}$	Size of the edge or central cloud storage resources	500 GB/2 TB per server
$N_{i}$	Number of iterations	300
$N_{p}$	Initial population number	50

## Data Availability

Not applicable.
